# Identification and characterization of the major pseudocoelomic proteins of the giant kidney worm, *Dioctophyme renale*

**DOI:** 10.1186/s13071-017-2388-x

**Published:** 2017-09-27

**Authors:** A. Nahili Giorello, Malcolm W. Kennedy, Marcos J. Butti, Nilda E. Radman, Betina Córsico, Gisela R. Franchini

**Affiliations:** 1 Instituto de Investigaciones Bioquímicas de La Plata (INIBIOLP), Facultad de Ciencias Médicas, Universidad Nacional de La Plata, La Plata, Argentina; 2 Institute of Biodiversity, Animal Health and Comparative Medicine, University of Glasgow, G12 8QQ, Glasgow, UK; 3 School of Life Sciences, University of Glasgow, G12 8QQ, Glasgow, UK; 4 Laboratorio de Parasitosis Humanas y Zoonosis Parasitarias, Cátedra de Parasitología Comparada, Facultad de Ciencias Veterinarias, Universidad Nacional de La Plata, La Plata, Argentina

**Keywords:** *Dioctophyme renale*, Lipid-binding proteins, Nematodes, Emerging zoonoses

## Abstract

**Background:**

The giant kidney worm, *Dioctophyme renale*, is a debilitating and potentially lethal parasite that inhabits and destroys, typically host’s right kidney, and may also be found in ectopic sites. It is circumglobally distributed, mainly in dogs, and is increasingly regarded as a threat to other domestic animals and humans. There is little information on the parasite’s true incidence, or immune responses to it, and none on its biochemistry and molecular biology.

**Results:**

We characterised the soluble proteins of body wall, intestine, gonads and pseudocelomic fluid (PCF) of adult parasites. Two proteins, P17 and P44, dominate the PCF of both male and females. P17 is of 16,622 Da by mass spectrometry, and accounts for the intense red colour of the adult parasites. It may function to carry or scavenge oxygen and be related to the ‘nemoglobins’ found in other nematode clades. P44 is of 44,460 Da and was found to associate with fatty acids by thin layer chromatography. Using environment-sensitive fluorescent lipid probes, P44 proved to be a hydrophobic ligand-binding protein with a binding site that is highly apolar, and competitive displacement experiments showed that P44 binds fatty acids. It may therefore have a role in distributing lipids within the parasites and, if also secreted, might influence local inflammatory and tissue responses. N-terminal and internal peptide amino-acid sequences of P44 indicate a relationship with a cysteine- and histidine-rich protein of unknown function from *Trichinella spiralis.*

**Conclusions:**

The dominant proteins of *D. renale* PCF are, like those of large ascaridids, likely to be involved in lipid and oxygen handling, although there is evidence of strong divergence between the two groups.

## Background

*Dioctophyme renale*, the giant kidney worm, is the largest known parasitic nematode of land vertebrates. It develops in, and completely destroys, mammalian kidneys it occupies, and is thereby a debilitating and potentially lethal parasite of dogs, domestic and wild animals, and humans. It is highly pathogenic and particularly common in dogs in South America [1–4], where it also severely affects wild animals such as bush dog (*Speothos venaticus*) [5], southern two-toed sloth (*Choloepus didactylus*) [6], crab-eating fox (*Cerdocyon thous*) [7] and maned wolf (*Chrysocyon brachyurus*) [8–10]. The latter is the largest native canid in South America and is considered as near threatened by IUCN (International Union for the Conservation of Nature - Red List 3.1), and dioctophymatosis is now included among threats to its wild population [11, 12]. Climatic change, environmental degradation, deforestation, and compromised sanitation around human settlements, have resulted in many biodiversity hotspots undergoing severe habitat fragmentation that has forced wild animals to migrate and face new threats, among which are increased vulnerabilities to *D. renale*. During such displacements, wild and feral species overlap in distribution with domestic animals, and potentially expose domestic animals and humans to sources of dioctophymatosis [13]. There are, for instance, sporadic reports of the infection in cattle, horses, pigs and rats [5, 14–16]. *Dioctophyme renale* is regarded as a causative agent of zoonotic disease in humans, in which the true incidence of exposure is unknown, though it has been an unequivocal direct cause of fatalities, or associated with renal carcinoma [17–20].

Adults of *D. renale* locate in the renal pelvis of one kidney (usually only the right one) of their definitive hosts, destroying the renal parenchyma and leaving a thin capsule enclosing a nest of mating parasites. The unaffected kidney usually hypertrophies to compensate for the complete loss of its counterpart’s function [8] such that there may be no overt clinical signs of infection for some time. Eggs are dispersed via urine into the environment, where they are thought to be ingested by an annelid (usually an oligochaete) in which the parasite’s larvae develop to the L3 stage [21]. If infected annelids are not consumed directly by a definitive host, the larvae can persist in paratenic hosts such as frogs, fishes, and rodents, and even a fresh water turtle [22].

*Dioctophyme renale* is globally distributed but is more common in South America than elsewhere. Dogs living close to rivers are commonly infected, as diagnosed by urine analysis, ultrasonography, surgery, or at necropsy [3, 4]. Although the parasite is usually located in one of the kidneys, infection can be bilateral, and worms may also develop to adulthood ectopically in sites such as the abdominal cavity, uterus, ovary, mammary gland, urethra, subcutaneous tissues of the inguinal region, and mesenteric lymph nodes [1, 23]. Recent studies have implemented a more sensitive technique for detecting and counting *Dioctophyme renale* eggs in urine [24]. Nevertheless, this and other currently used diagnostic methods for dioctophymatosis may fail to detect premature, infertile, single sex, or ectopic infections, and the equipment required makes screening for the infection in rural and economically deprived areas difficult. Recently published data, however, indicate that soluble antigens from the digestive tract of *D. renale* may provide the basis of a serodiagnostic method for dioctophymatosis in dogs [25]. A serodiagnostic method applicable to humans is also urgently required given that people living in poverty, poor sanitary conditions, and of poor sociological backgrounds, may consume intermediate hosts and become infected as well as contributing to the persistence of the parasite by feeding their dogs with uncooked material from the same paratenic hosts.

Despite the importance and threat that dioctophymatosis poses to humans, domestic, and endangered wild animals, there is a paucity of molecular and immunological information on this parasite. We here begin to address this neglect with a report on a comparison of the protein profiles of its tissues, concentrating on the most readily available source of the parasite’s soluble proteins, its pseudocelomic body fluid (PCF). We then concentrated on the two most abundant PCF proteins that occur in both males and females with a view to establishing their biological functions.

## Methods

### Parasite material

Nematodes were isolated by nephrectomy of affected dogs diagnosed in surrounding areas of La Plata, Argentina. Freshly-collected parasites were washed several times with phosphate buffered saline pH 7.4 (PBS) and placed in a Petri dish. A small incision was made close to the anterior end of adults worms using a scalpel and PCF was allowed to drain freely into the Petri dish, filtered (0.22 μm pore size) and stored at -20 °C. The rest of the parasites’ bodies were dissected into body wall (BW), intestine (I) and gonads (testis, T; ovary, O), which were homogenized using a mechanical tissue grinder. All samples were in PBS buffer containing a cocktail of protease inhibitors (Protease Inhibitor Cocktail Set III Calbiochem cat. 539,134, Merck Millipore, San Diego, USA) and stored at -20 °C. Protein content was estimated using the Bradford assay [26].

### Polyacrylamide gel electrophoresis (SDS-PAGE)

Protein gels (1 mm thick, 6% stacking, 15% acrylamide final separating) were run in a Miniprotean II (Bio-Rad, Hercules, USA) slab gel apparatus. Samples were heated in a heating block set for 100 °C, with 5% β-mercaptoethanol added for reducing conditions. Following electrophoresis, gels were stained with Coomassie Brilliant Blue R (Sigma-Aldrich, B0770, Saint Louis, USA) overnight, and destained using a 30% ethanol/10% acetic acid/60% water v/v/v solution. Apparent molecular masses were estimated by mobility relative to standard marker proteins (GE Healthcare UK Limited GE; LMW 17–0446-01, Amersham Place, Buckinghamshire, UK), and are indicated in kilodaltons (kDa).

### Size exclusion chromatography

Size exclusion chromatography (SEC) was carried out using an Âkta FPLC System (GE Healthcare Life Sciences, Uppsala, Sweden) to purify the major proteins from PCF and to indicate their aggregation state. Briefly, 400 μl of filtered PCF were loaded onto a Superdex 75 HR 10/30 column (GE Healthcare Life Sciences, Uppsala, Sweden) pre-equilibrated with PBS. A flow rate of 0.7 ml/min was used, and samples were collected using an inline F900 fraction collector. The column was pre-calibrated using bovine serum albumin (66 kDa), carbonic anhydrase (29 kDa) and cytochrome *c* (12 kDa) as protein standards under the same chromatography conditions, and molecular size estimations of the proteins were undertaken as described [27]. Samples, as well as standard proteins, were analysed twice under the same conditions to ensure reproducibility. Elution profiles were recorded following the UV absorption at 215 and 280 nm.

### Lipid-binding activity in native polyacrylamide gels

In order to identify lipid-binding proteins present in PCF, samples were pre-incubated with the environment-sensitive fluorescent probe 11-[5-(dimethylamino)-1-naphthalenesulfonylamino] undecanoic acid (DAUDA, Invitrogen Molecular Probes, Eugene, USA). One μl of a 60 μM DAUDA solution was added to 20 μl of PCF at a concentration of 6–10 μg/μl and incubated for 30 min at 20 °C. As a positive control, a well-characterised fatty acid and retinol-binding protein of nematodes [28–30] (recombinant ABA-1A from *Ascaris suum*) was also included. Samples pre-incubated with DAUDA were then resolved in 15% gels without addition of SDS, reducing agent, or heating. Without any further staining, fluorescence emission was visualised using an Image Quant 350 (28–9272-95, GE Healthcare Life Sciences, Uppsala, Sweden), using the UV light as excitation light source and a fluorescein filter set (537/35 nm) for emission detection. After the fluorescence image was captured a final step of gel staining with Coomassie Brilliant Blue R was carried out on the same gel.

### Lipid extraction and analysis

Total lipids were extracted from PCF or purified proteins as previously described [31, 32], with minor modifications. PCF or protein fractions (0.5 ml) were mixed with 5 ml CHCl_3_:CH_3_OH (2:1 v/v) and vigorously shaken for 15 min in an ice bath. The homogenate was stored overnight at -20 °C and the next day 250 μl of 2.9% w/v NaCl solution was added and vortexed. The aqueous and solvent phases were allowed to separate at room temperature and by a centrifugation step. The upper, aqueous phase, was discarded and the lower phase containing lipids was recovered and dried under a stream of nitrogen gas, re-dissolved in CHCl_3_ and stored at -20 °C. Lipid classes found in PCF samples were resolved by thin layer chromatography (TLC) using previously described methods and solvent systems by [32]. Lipid samples obtained from PCF along with lipid standards were spotted onto TLC plates (20 × 20 cm; previously activated at 100 °C for 30 min) and developed with methyl acetate/ isopropanol/ chloroform/ methanol/ 0.25% KCl (25:25:25:10:9, by volume) for polar lipids. Rat liver lipids previously obtained in our lab were included as reference. Plates were developed using the sulfuric acid charring method. Briefly, plates were sprayed with a 8% CuSO_4_ (w/v), 10% H_3_PO_4_ (v/v) solution and the lipids visualized as black carbon depots formed after heating the plates at 180 °C for 1 h.

### Spectrofluorimetry

Fluorescence experiments were performed with a Fluorolog-3 Spectrofluorometer (Horiba-Jobin Yvon, Edison, NJ). Buffer alone was used to correct for Raman scattering where stated. Two environment-sensitive fluorescence probes were used, the non-specific hydrophobic probe 8-anilinonaphthalene-1-sulfonic acid (ANS) and the fluorophore-tagged fatty acid 11-[5-(dimethylamino)-1-naphthalene sulfonylamino] undecanoic acid (DAUDA; see above). The latter is considered to be more selective of dedicated lipid-binding sites in proteins and the fluorescence emission spectrum of its dansyl fluorophore is taken to indicate the degree of apolarity of its environment [33]. To detect lipid-binding activity among proteins of PCF, 5 μl of a 60 μM DAUDA stock solution in ethanol were added to 200 μl of each fraction after size exclusion chromatography to monitor lipid-binding activity. Spectra were recorded using λ_exc_ = 345 nm, λ_em_ = 360–650 nm. DAUDA stock solutions were prepared in ethanol and then diluted in PBS for use in the assays. After SEC purification P44 was delipidated using reverse phase high performance liquid chromatography protocol as described previously [29, 34] and employed in all ligand-binding experiments. For ANS, the excitation wavelength was 350 nm and fluorescence emission was collected in the range 420–600 nm. Intrinsic fluorescence of protein was measured in presence and absence of oleic acid, excitation wavelength was set to 295 nm and emission was collected in the range 310–400 nm. In all of the above case, the slit widths were set to 5 nm for both excitation and emission. All emission spectra were plotted and analysed using Microcal ORIGIN software.

### Protein mass spectrometry and N-teminal amino acid sequencing

Mass spectrometric analyses and N-terminal sequencing were carried out at the National Laboratory of Peptide and Protein Research (LANAIS PROEM, School of Pharmacy and Biochemistry, University of Buenos Aires) in a MALDI TOF TOF 4800 plus mass spectrometer (ABSciex, Framingham, MA). N-terminal sequencing was performed in a PPSQ-31A (Shimadzu Corporation, Japan). Direct N-terminal sequencing by Edman degradation provided sequences for 18 and 19 amino-acids for P17 and P44, respectively. For P44, additional peptides were obtained following digestion with trypsin, Glu-C proteinase or Lys-C proteinase. Blastp searching of the nematode databases yielded no similar proteins for the P17 sequence. For P44, one pair of peptides overlapped, and another pair were also found to do so in preliminary searches. With these peptides edited together, there was a convincing fit with the amino acid sequence of a poly-cysteine and histidine-tailed protein isoform 2 from *Trichinella spiralis* (NCBI accession gb|AEQ29641).

## Results

### Somatic and pseudocoelomic fluid (PCF) proteins of adult *D. renale*

**Fig. 1:**
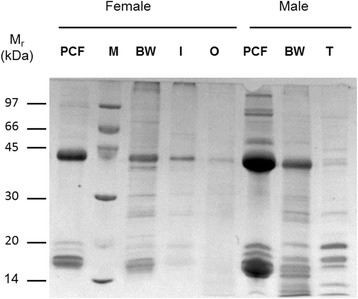
Protein profiles of tissues and pseudocoelomic fluid (PCF) of adult *D. renale.* SDS-PAGE of soluble protein extracts from the PCF and tissues of adult female and male adult worms run under reducing conditions and stained with Coomassie Blue. Lane PCF: pseudocelomic fluid; Lane BW: body wall; Lane I: intestine; Lane O: ovary; Lane T: testis; Lane M: reference proteins with masses given in kiloDaltons (kDa). Other gel analysis showed that the protein profile of male and female intestines were similar (not shown). *Abbreviation*: M_r_, relative mobility

PCF and soluble proteins of body wall, intestine, testis, and ovary were separated by SDS PAGE (Fig. 1). The protein profile of PCF is dominated by two notably abundant proteins, one of M_r_ 17,000 (designated P17) and another of M_r_ 44,000 (P44) in both males and females. The proteins of body wall, intestine, testis, and ovary showed little overlap with each other except for the common presence of a protein of similar M_r_ to P44. The protein profile of *D. renale* PCF is different from those of the only other parasitic nematodes large and accessible enough for PCF to be recovered easily, namely the adults of large ascaridids such as *Ascaris suum* and *Toxocara canis* [35, 36]. The most abundant proteins in adult ascaridid PCFs are approximately 14.4 kDa monomers of the nematode polyprotein allergens (NPAs) [30, 37, 38], and a globin of approximately 43 kDa [39, 40]. A recombinant form of the NPA from *Ascaris suum*, ABA-1A (syn. As-NPA-1A; [29]), was included here as a positive control, and there was no sign of an abundant protein of similar size in *D. renale* PCF (not shown).

### Purification of P17 and P44 by gel filtration

**Fig. 2:**
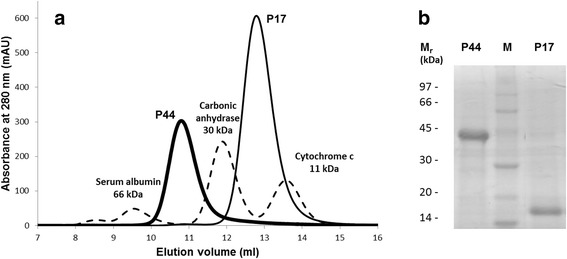
Purification of the abundant P17 and P44 proteins of adult *D. renale* pseudocoelomic fluid. **a** Pure major gel filtration peaks of *D. renale* PCF correspond to the P17 and P44 proteins (solid lines), mass spectrometry of which yielded masses of 16,622 Da and 44,460 Da, respectively (see main text). Absorbance peaks from the three standard proteins are overlain (dashed lines). **b** SDS-PAGE analysis of the different fractions after gel filtration. Coomassie Blue-stained. Lane M: reference proteins with masses given in kiloDaltons (kDa). *Abbreviation*: M_r_, relative mobility

PCF was fractionated by size exclusion gel chromatography through a Superdex 75 column (Fig. 2a). The two major proteins were readily separable, as seen from the accompanying SDS-PAGE of the resulting fractions (Fig. 2b). The sizes of these proteins as calculated relative to the time of elution of standard calibration proteins approximated the values obtained from SDS-PAGE under reducing conditions (Fig. 1), indicating that both proteins eluted from the column as monomers, and may therefore exist as such in PCF. Protein mass spectrometry of the two provided molecular masses of 44,460 Da for P44 and 16,622 Da for P17 (data not shown). The P17 protein is intensely red, and retains its colour in gel filtration and in native non-reducing PAGE (see below), but not under SDS-PAGE. The red chromophore is probably, therefore, non-covalently attached, as is the haem prosthetic group in proteins such as vertebrate globins (haemoglobin, myoglobin, neuroglobin). P17’s molecular mass is similar to that of the ‘nemoglobins’ that have been described from nematodes [41], but which are not related to vertebrate globins. The nematodes from which nemoglobins have been described are in clades (Clades II to V) that are phylogenetically remote from Clade I in which *D. renale* is now placed [42, 43], so P17’s evolutionary relationship, if any, to nemoglobins remains to be established.

### Protein-associated lipids in *D. renale* PCF

**Fig. 3:**
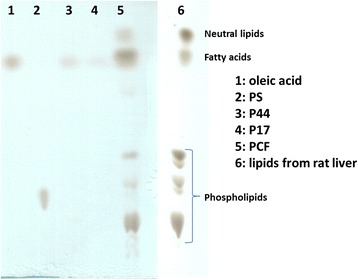
Lipids associated with the abundant P17 and P44 proteins of *D. renale* pseudocoelomic fluid. Lipids were extracted from purified P44, P17 or unfractionated PCF as described in Methods, and separated by TLC to resolve polar lipids. Rat liver lipids were included for comparison. PCF contains similar lipids to rat liver but P44 appeared to associate selectively with fatty acids. P17 shows trace association with fatty acids but does not bind DAUDA (see main text)

**Fig. 4:**
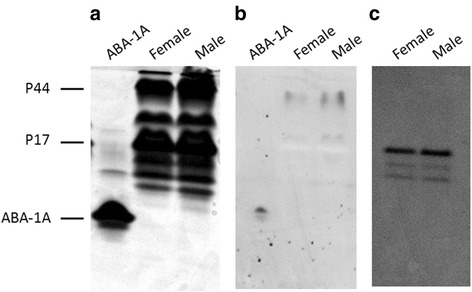
Lipid-binding by proteins in *D. renale* pseudocoelomic fluid. **a** Native (non-SDS) polyacrylamide gel electrophoresis of adult worm PCF and the fatty acid-binding recombinant ABA-1A of *Ascaris suum* as a control. Coomassie Blue stained gel. **b** The same as **(a)** gel in which protein samples were pre-incubated with the environment-sensitive fluorescent fatty acid analogue DAUDA before electrophoresis, viewed under ultraviolet light, and the negative image of DAUDA’s fluorescence emission is shown. Proteins migrating commensurate with P44 and ABA-1A in size associate with DAUDA indicative of lipid-binding activity. **c** Similar, unstained gel viewed under visible light is which only the intensely red P17 is visible. P17 loses its colour under SDS-PAGE (not shown), indicating that the red chromophore is not covalently bound. Comparison with **(b)** shows that P44, but not P17, binds DAUDA, which was also found to be the case in spectrofluorimetry (see main text)

The most abundant proteins found in animal blood or haemolymph are often respiratory or lipid-carrier proteins. In order to determine the lipid classes present in PCF and associated with P17 or P44, Folch lipid extractions from PCF and the two purified proteins were carried out and analysed by TLC. As seen in Fig. 3, the lipids found in unfractionated PCF were heterogeneous and similar in type and relative concentrations to those of rat liver. In contrast, lipids found in association with P44 (and to a minor extent with P17) were fatty acids. As an additional screen, PCF was incubated with DAUDA [an 11-carbon fatty acid tagged at its omega (methyl) end with the environment-sensitive dansyl fluorophore; see Methods for details] prior to loading onto non-denaturing PAGE (Fig. 4). There would have been some loss of protein-bound DAUDA during electrophoresis because it should not have bound covalently, but a protein migrating to the same position as P44 was observed to bind the probe, as also did the ABA-1A positive control protein, but not P17 (compare Fig. 4a, b). Figure 4c shows a similar non-denaturing PAGE of PCF that had not been stained, as photographed under visible light. P17 retained its red colour under these conditions; a strong red band appeared at the M_r_ for P17, but also two clear bands of lower M_r_ that may indicate other isoforms or charge variants of the protein.

### Spectrofluorometric analysis of lipid-binding by P44

**Fig. 5:**
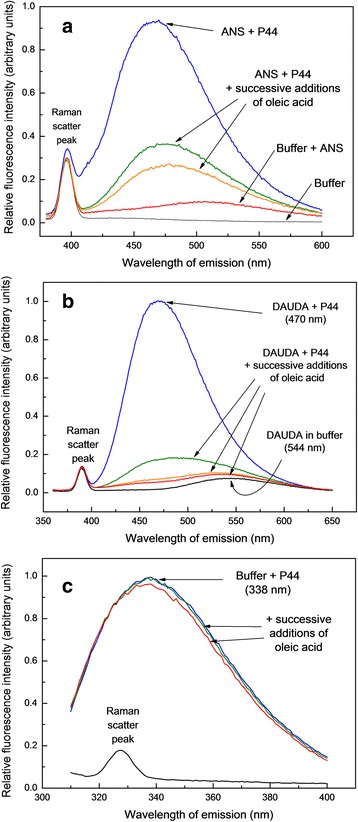
Hydrophobic ligand-binding by *D. renale* P44. **a** Superdex 75-purified and delipidated P44 (1.3 μM) was added to the non-specific hydrophobic probe ANS (3.3 μM) in PBS causing a dramatic increase in the probe’s fluorescence emission. Successive additions of 0.5 μl of 1 mM oleic acid partially reversed the fluorescence enhancement. Excitation wavelength = 350 nm. **b** P44 (1.3 μM) added to the dansyl fluorophore-tagged fatty acid DAUDA (0.3 μM) in buffer yielding a dramatic increase in fluorescence intensity and blue shift in DAUDA’s peak wavelength of emission from 544 nm to 470 nm. This was reversed with successive additions of 0.5 μl of 1 mM oleic acid solution. Similar experiments using *D. renale* P17 revealed no binding of DAUDA (not shown). Excitation wavelength = 345 nm. **c** Intrinsic (predominantly tryptophan) protein fluorescence emission by P44. P44 in buffer was excited at 295 nm producing peak fluorescence emission at 338 nm. Successive additions of 0.5 μl of 1 mM oleic acid solution in ethanol to 300 μl protein in PBS produced only very slight if any changes in emission intensity and wavelength of peak emission. The spectrum of buffer alone (shown) was subtracted from each of the P44 intrinsic fluorescence emission spectra

The PCF of other large nematodes, such as adult ascaridids, have high concentrations of lipid-binding proteins, particularly the NPAs mentioned above. Given that fatty acids associated with P44 (Fig. 3), we further characterised the ligand-binding properties of the protein using environment-sensitive fluorescent probes and P44 from which any resident lipid had been removed by reverse-phase HPLC performed as previously described [34]. We first used 8-anilinonaphthalene-1-sulfonic acid (ANS), a non-specific probe of hydrophobic sites in proteins, which exhibited a dramatic enhancement of fluorescence emission in the presence of P44 (Fig. 5a). Addition of oleic acid to preformed P44: ANS complexes resulted in displacement of ANS from the protein. We then used DAUDA, the environment-sensitive dansyl fluorophore that exhibits low florescence in water and that is markedly enhanced and blue-shifted in emission when in an apolar environment such as a protein binding site [44]. This probe binds to some but not all specialised fatty acid-binding proteins of mammals, and also to the NPAs and other lipid-binding proteins found only in nematodes (nematode fatty acid and retinol-binding proteins (FARs), and nemFABPs; [45–47]). DAUDA’s fluorescence emission was dramatically enhanced when mixed with de-lipidated P44, and its peak wavelength of emission underwent a dramatic blue shift from 544 nm to 470 nm (Fig. 5b), which was similar to that seen with the well-characterised ABA-1 used as a control (not shown) [48]. Blue-shifts in DAUDA of this magnitude are rarely seen with, for instance, cytosolic fatty acid-binding proteins [48, 49] or vertebrate serum albumins [44], and is indicative of a highly apolar binding site environment. When oleic acid was added to preformed DAUDA:P44 complexes there was a clear displacement of the probe from the protein, which is indicative of the binding site being preferential for fatty acids and that the attached dansyl fluorophore is probably irrelevant to binding.

In some lipid-binding proteins the binding of a ligand alters the environment of a tryptophan (Trp) side chain that is detectable by a change in the intensity and/or wavelength of fluorescence emission by the Trp [50]. We do not yet know the complete amino-acid sequence of P44 but excitation of a sample of the de-lipidated protein at 295 nm (at which fluorescence emission by tryptophan dominates over that by phenylalanine and tyrosine) elicited an emission spectrum shown in Fig. 5c. With the Raman scatter of buffer subtracted, the peak wavelength of intrinsic florescence emission by P44 occurred at approximately 338 nm. The fluorescence emission of Trps is sensitive to the polarity of the environment of its indole side chain such that full exposure to solvent water will yield an emission maximum at about 356 nm, which is blue-shifted in proteins in which a Trp is buried [28, 29, 51]. In the case of P44, the wavelength of maximum emission is 338 nm (Fig. 5c), representing a blue shift of about 18 nm from that of a Trp that is fully exposed to solvent water (e.g. [28, 52]. We do not yet know the exact number of Trps in P44 so the emission could come from more than the four already found by N-terminal sequencing (see below). The emission spectrum will therefore be a composite of emissions by two or more Trps that may have differing environments, but P44’s intrinsic emission spectrum indicates the isolation of some at least of the protein’s Trps from solvent water. If the environment of a Trp side chain is affected by protein binding to a lipid ligand then the emission spectrum may be further blue-shifted or increased in intensity [50]. With P44, however, the addition of oleic acid to de-lipidated protein did not cause a significant change in fluorescence emission intensity or peak wavelength, so it may be that none of the protein’s Trps are close to or in the binding site, or affected by ligand-binding causing environmental change elsewhere in the molecule. Another possibility could be that a single Trp’s environment is affected by lipid-binding to the protein, but that its altered emission spectrum is swamped by emission form other Trps that are not affected.

### Amino-acid sequence analysis

**Fig. 6:**
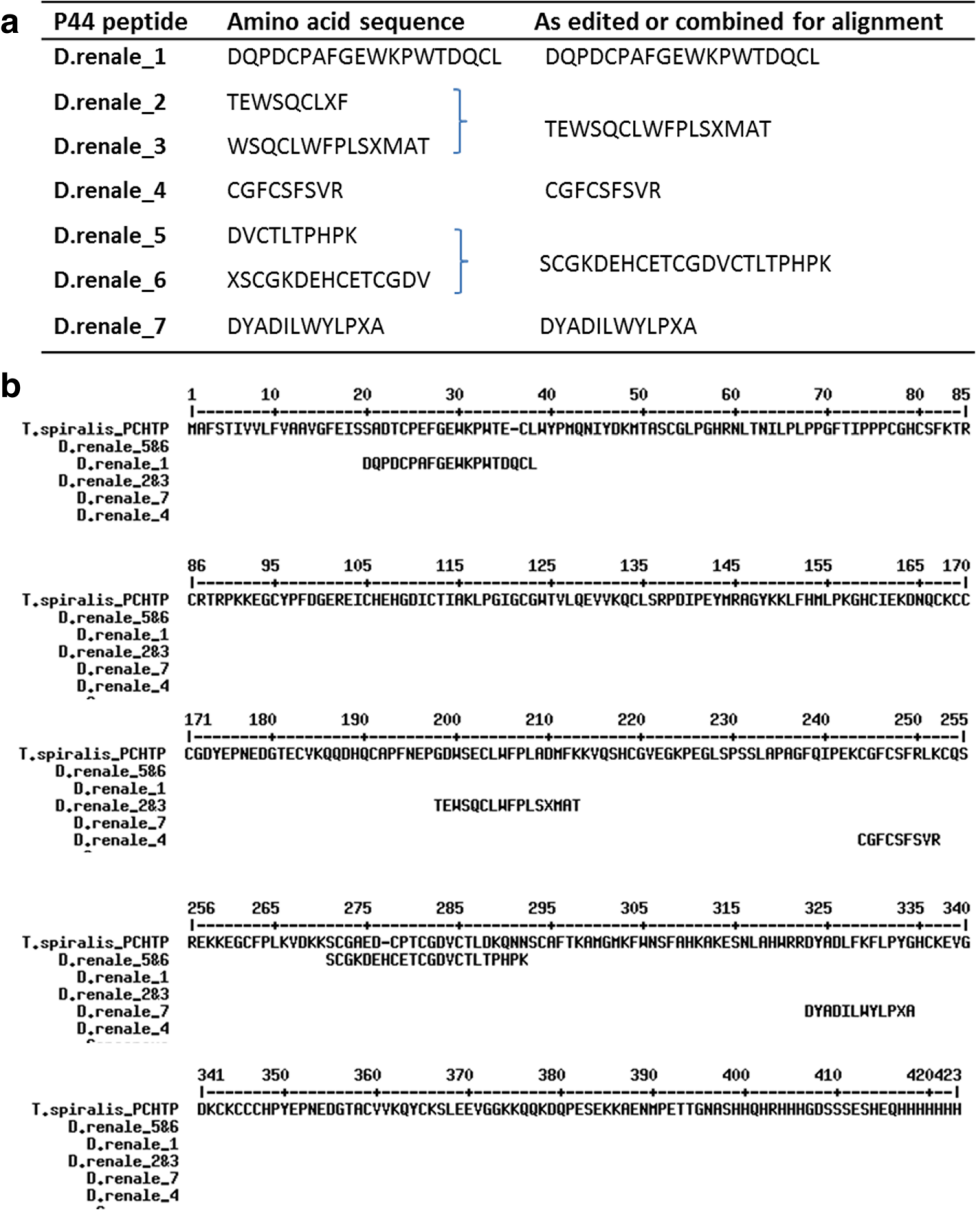
Putative orthologues of *D. renale* P44. **a** Peptide sequences from P44 and how they were combined or edited for the alignment. Sequences are given using the single letter code for amino-acids. **b** Alignment between the peptides obtained from P44 and the full predicted amino-acid sequence of the poly-cysteine and histidine-tailed protein isoform 2 from *Trichinella spiralis* (Ts-PCHTP; NCBI accession AEQ29641). The alignment was made using MultAlin set for the default blosum62 substitution matrix (http://multalin.toulouse.inra.fr/multalin/multalin.html). Though not shown, the alignment was similarly good with sequences from other species of *Trichinella*, and with a PCHTP sequence from *Trichuris trichiura* (NCBI accession CDW54015). The first 21 amino-acids in the *T. spiralis* sequence are predicted to be a cleavable signal peptide by SignalP (www.cbs.dtu.dk/services/SignalP/), the predicted mature protein therefore beginning close to where the *D. renale* P44 N-terminal peptide sequence begins (D.renale_1)

From its size and ligand-binding characteristics, P44 appears to be a novel type of lipid-binding protein of nematodes. The blue shift in DAUDA emission upon binding is similarly extreme to that seen in the NPA and FAR proteins of nematodes [28, 47], but the post-translationally processed forms of the former are approximately 14.4 kDa (although some species have larger combined units [53] and the FAR proteins are all about 20 kDa in size [34, 54, 55]. To investigate whether P44 exhibits any relationship to known proteins from nematodes, we obtained N-terminal and several internal peptide sequences (Fig. 6). Database searching revealed that the peptide sequences all align reasonably well with the sequence of a protein from *Trichinella spiralis* that is described as a poly-cysteine and histidine-tailed protein (PCHTP; Fig. 6) [56]. The similarities are collectively convincing given the alignments of cysteines and bulky hydrophobic amino acids such as tryptophan and phenylalanine. A similar degree of similarity was found with PCHTPs predicted for other species of *Trichinella* and also *Trichuris trichiura,* all of which are, like *D. renale,* Clade I nematodes. An N-terminal amino acid sequence was also obtained from P17 (TQNKPLLTAQMDXIHADA; single amino-acid code), but alignments even with putative globin-like proteins from Clade I nematodes (*Trichinella* and *Trichuris* species) in current databases were not convincing.

## Discussion

We present a protein biochemical analysis of the giant kidney nematode *D. renale*. Species of animal-parasitic nematode are scattered across the Phylum Nematoda, but those in Clade I, to which *D. renale* belongs [43]. The Dorylaimia, have received little attention except for those in the genera *Trichinella* and *Trichuris*, representatives of which are the subjects of intense immunological and molecular biological research. We have begun work on *D. renale* because of its veterinary and potential human medical importance, but also because of its phylogenetic position within a relatively under-studied clade of nematodes. The sheer size of the adult worms presents a significant advantage for investigating their molecular and cellular components, equalled elsewhere only by the ascaridids of Clade III. This allowed us to isolate different tissues from both males and females, and their PCF can be readily extracted to provide plentiful quantities of its constituent proteins. In that PCF we found two proteins that were particularly abundant, one that confers the intense red colour of the adult parasites and may be haem-containing, and the other a potentially novel type of lipid-binding protein.

The protein profile of the PCF of adult *D. renale* (Clade I) males and females is completely different from that of the equivalently-sized *Ascaris* and *Toxocara* spp. of clade III [35]. The last common ancestor of the two clades is thought to have lived between 500 and 650 million years ago [57]. The major proteins in PCF of these large gut-dwelling ascaridids are the lipid-binding NPAs (e.g. *aba*-1 of *Ascaris*) [28, 38], but we found no indication that they are present in *D. renale*. This was somewhat unexpected given that this family of proteins has been found in species of nematode of clades III, IV and V [58]. Also, to date, we have found no evidence yet of NPA-encoding genes in the genome databases for *Trichinella spiralis* or *Trichuris* spp. It is therefore possible that the NPAs either appeared as novelties in the common progenitor of the Rhabditida (Clades III, IV and V; little is known of the biochemistry of Clade II nematodes), or that they were lost in Clade I.

Nematodes produce and secrete an unexpectedly wide range of unusual lipid-binding protein types, many of which are structurally distinct from those of their hosts, and which have been recognised as major antigens in infection [59, 60]. The acquisition and transport of lipids is crucial to these organisms because of their inability to synthesise complex lipids [61, 62], and, in the case of large parasites, they need to distribute insoluble lipids between the site of absorption to the tissues where they are consumed. Hypothetical roles for helminth lipid-binding proteins include internal functions common to most multicellular organisms, such as distribution of energy stores and for cell-cell signalling in development. The secretion of lipid-binding proteins by parasites may be additionally involved in interference with, and manipulation of, a host’s lipid-based cellular or immune functions [63, 64].

The NPAs and FAR proteins of nematodes have no recognizable structural counterparts in other animal groups [59, 60]. While there is much yet to establish, it is possible that P44 represents another novel class of lipid-binding protein that may be specific to nematodes. The protein with which we found amino-acid sequence similarities to P44 is an unusual poly-cysteine and histidine-tailed protein (PCHTP) from species of the genera *Trichinella* (and *Trichuris;* Fig. 6). Radoslavov et al. [56] stated that they found the *T. spirals* protein Ts-PCHTP to interact with DAUDA but not convincingly so. That could be due to a misfolding of the recombinant form of the protein that was used given its unusually high number of cysteines, potentially leading to a high proportion of incorrectly disulphide-bonded protein. Also, it might be necessary to delipidate the protein for DAUDA binding to be discernible. Interestingly, these PCHTPs are unusually rich in histidines, which is also true of some units of the NPAs, in which the histidine-rich peptides have been found to bind zinc ions (M.W. Kennedy and A. Cooper, unpublished). For the moment, though, P44 now joins the select group of proteins, all specific to nematodes, that induce the greatest blue shift in peak fluorescence emission of the environmental-sensitive probe DAUDA, which is greater than that observed with specialist lipid-binding proteins of vertebrates such as cytoplasmic fatty acid binding proteins and serum albumins [44, 46, 48].

NPAs appear to be the major lipid carriers in the PCF of large adult ascaridids (*Ascaris* spp. and *Toxocara canis*), and have been reported in many species in Clades III, IV and V [28, 48]. NPAs are synthesized as large tandem arrays of units that have similar amino-acid sequences and lipid-binding properties that are posttranslationally cleaved down to multiple copies of about 14.4 kDa units [28, 48, 65]. In vertebrates, the counterpart lipid carriers in blood would be the serum albumins, which are about 67 kDa, monomeric, carry fatty acids and a range of other apolar and polar ligands, and perform an important osmotic function in blood. Serum albumin itself is derived from gene duplication events [66] to create a protein of a size that will not be lost through the excretory system’s size filter of vertebrate kidneys. A surprise, therefore, is that the single, small, highly abundant units of the NPAs are not lost from a nematode’s PCF unless there is a low molecular size filter in their excretory systems, or a recovery system to avoid loss. This problem may also apply to P17 of *D. renale*, but possibly not to its P44 because of its larger size. While we find strong evidence that P44 is an unusual type of lipid carrier protein, it remains to be established whether it also has other transporter functions.

The P17 protein of *D. renale* is intensely red, abundant in PCF, and must account for the dramatic red colour of the worms. It is probably haem-containing, in common with many respiratory proteins in animals, and the red colour is indicative of it having iron at its core [67]. P17 may therefore be a member of a class of nematode-specific proteins termed “nemoglobins” to distinguish them from the unrelated haemoglobins of vertebrates (although the term “haemoglobin” still persists for these proteins in the nematode literature [41]). The precise function of nemoglobins is still debated but they are likely to be oxygen transporter proteins similar to the haemoglobins and myoglobins of vertebrates, although they may also serve to sequester oxygen in species that are micro- or anaerobic [41, 68]. We obtained N-terminal amino acid sequence from P17, but we have not yet found any similar proteins in the nematode databases.

As noted above, proteins from the “oesophagus” of adult *D. renale* provide improved target antigens for serological detection of dogs infected or exposed to this parasite, including those that are not releasing parasite eggs through their urine [25]. It will be interesting to establish whether the parasite’s PCF is of equivalent value, especially given the relative ease with which it can be obtained in quantity from adult parasites. We do not as yet know whether the tissue-migratory pre-adult stages of *D. renale* produce all or any of the PCF proteins we observed in adults, and understanding the developmental protein expression pattern of the parasite would clearly be valuable in designing an effective immunoassay for detecting pre-adult, single sex, and ectopic infections.

In general terms this work is aimed at discovering species-specific proteins from *D. renale* that may be useful as new diagnostic markers for use in dogs and humans, and also potential new immune- or chemo-therapeutic targets. The development of diagnostic methods of enhanced specificity and sensitivity will also contribute to improved understanding of the epidemiology of *D. renale*. Additionally, our results may not only bring to light LBPs from parasitic helminths that may be structurally novel, but could also reveal new aspects of lipid metabolism of parasitic nematodes, a barely explored area in Clade I nematodes. The functional data that may emerge from this work could also contribute to a better understanding of the host-parasite relationship in this unusual infection. Analysis of P44 in particular has also emphasised the closeness of *D. renale* to other highly pathogenic parasitic members of Clade I nematodes, and may provide valuable insights to the lipid handling systems of parasites such as *Trichinella* and *Trichuris* species.

## Conclusions

In this work our findings begin to illuminate the biology of this unusual parasite at the molecular level. We have described two major proteins in the PCF: P44, which was revealed to exhibit lipid-binding activity, and P17, a possible “nemoglobin”. These findings are also of importance given that they provide material and information pertinent to the function of the components of smaller related species.
